# IgG4 related lung disease mimicking multiple pleural disseminated lung cancer

**DOI:** 10.1002/rcr2.1044

**Published:** 2022-09-27

**Authors:** Koya Ito, Yukiko Maeda, Akane Mita, Saori Aizawa, Arei Mizushima, Natsuko Taniguchi, Katsura Nagai, Hiroki Shomura, Atsuo Hattori, Toshiyuki Harada

**Affiliations:** ^1^ Center for Respiratory Diseases Japan Community Healthcare Organization Hokkaido Hospital Sapporo Japan; ^2^ Department of Surgery Japan Community Healthcare Organization Hokkaido Hospital Sapporo Japan; ^3^ Department of Pathology Japan Community Healthcare Organization Hokkaido Hospital Sapporo Japan

**Keywords:** IgG4, IgG4 related lung disease, immunologic abnormality, lung cancer, subpleural pulmonary nodule

## Abstract

IgG4‐related lung disease (IgG4‐RLD) can present with various types of radiological findings such as nodule, ground‐glass opacity, alveolar interstitial, and bronchovascular involvement. IgG4‐RLD generally manifests as mild clinical symptoms, despite evidence from the image findings. Herein we report an asymptomatic patient with IgG4‐RLD mimicking multiple pleural disseminated lung cancer.

## Graphical abstract

Herein we report an asymptomatic patient with IgG4‐related lung disease mimicking multiple pleural disseminated lung cancer.

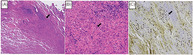

## CLINICAL IMAGE

**FIGURE 1:**
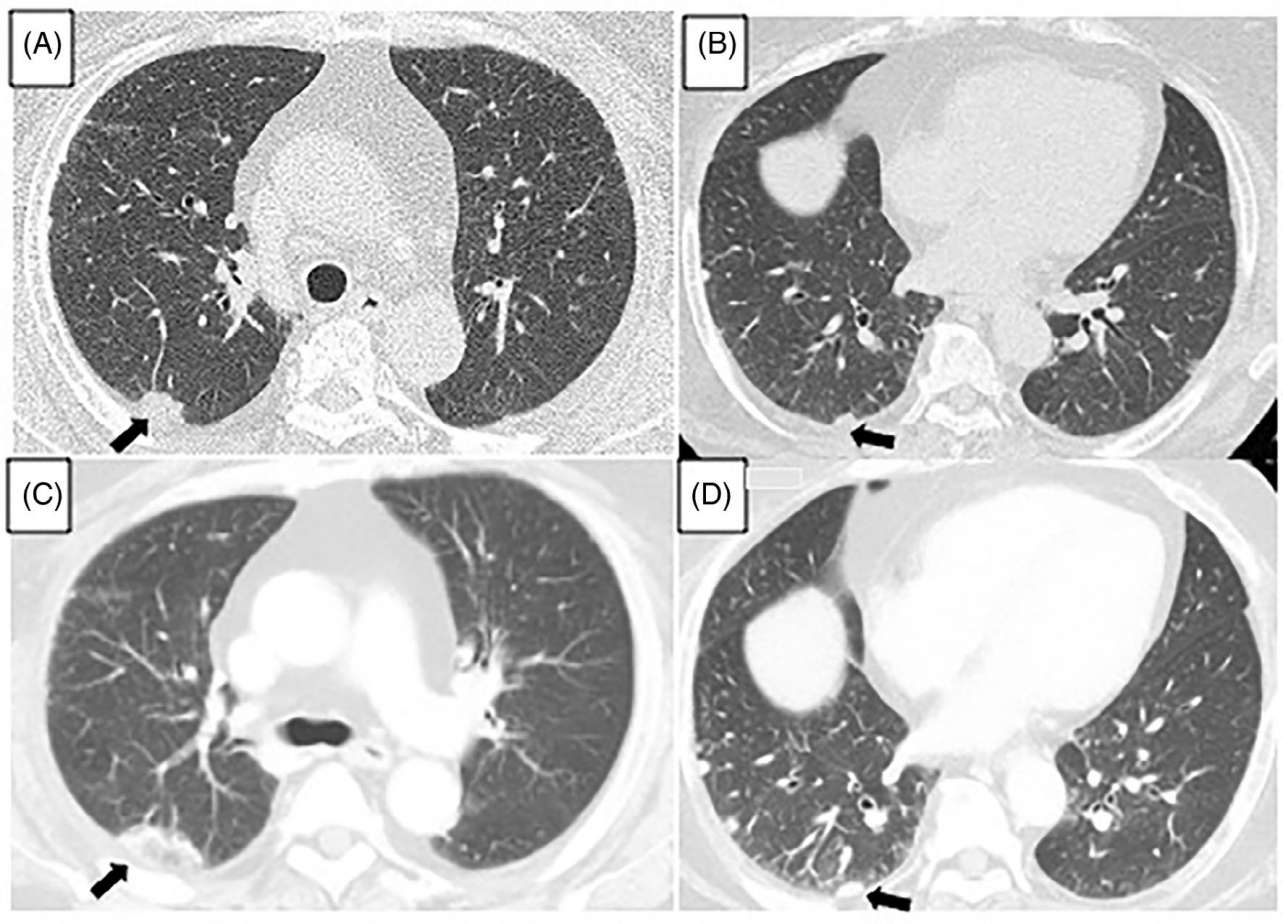
Chest CT scans showing an increase in the size and number of subpleural pulmonary nodule (A, B: at first visit; C, D: in the subsequent visit after 1 month). Arrows indicate subpleural pulmonary nodule

**FIGURE 2:**
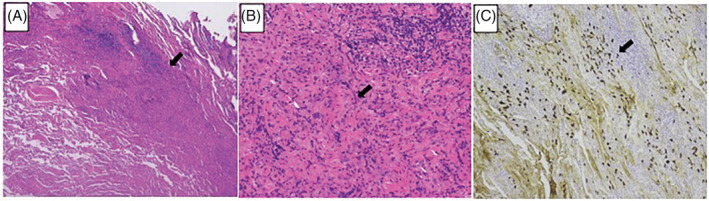
Histopathological findings of a surgically resected specimen with haematoxylin and eosin staining (A, B) and IgG4 staining (C). Arrows indicate dense lymphoplasmacytic infiltration (A), storiform fibrosis (B), and a 100% IgG4/IgG ratio (C)

A 68‐year‐old woman, a 30‐pack‐year smoker with a medical history of diabetes and hypertension, presented to our department for assessment of multiple subpleural pulmonary nodules detected through chest computed tomography (CT). The laboratory examinations including tumour markers, infection, and vasculitis were unremarkable. One month later, the nodules rapidly grew in size and number (Figure 1), then primary lung cancer with pleural dissemination was suspected. Histopathological findings of the surgically resected nodules revealed lymphoid follicular hyperplasia with dense lymphoplasmacytic infiltration and storiform fibrosis. Numerous IgG4‐positive plasma cells were observed (Figure 2). These findings confirmed the diagnosis of IgD4 related lung diseases (IgG4‐RLD). Fluorodeoxyglucose‐positron emission tomography revealed no extra‐thoracic lesions. Because the patient was asymptomatic and the disease was stable, no specific treatment was indicated. We plan to monitor the patient and conduct periodic physical examinations, laboratory testing, and surveillance CT. As IgG4‐RLD can present with various types of radiological findings,^1^ IgG4‐RLD is often misdiagnosed as lung cancer. IgG4‐RLD generally manifests as mild clinical symptoms, despite the degree of lesions and their spread, determined in the imaging analysis, and excessive cellular infiltration, observed during pathological examinations.^2^ Clinicians should keep IgG4‐RLD in mind as a potential diagnosis for asymptomatic multiple pleural nodules.

## AUTHOR CONTRIBUTIONS

All authors contributed to the study conception. The literature search was performed and the first draft of the manuscript was written by Koya Ito and Toshiyuki Harada. Yukiko Maeda, Akane Mita, Saori Aizawa, Arei Mizushima, Natsuko Taniguchi, and Katsura Nagai contributed data curation and formal analysis. Hiroki Shomura performed surgical resection. Atuso hattori contributed the pathological diagnosis. All authors commented on previous versions of the manuscript. All authors have prepared, read and approved the final manuscript.

## CONFLICT OF INTEREST

None declared.

## ETHICS STATEMENT

The authors declare that appropriate written informed consent was obtained for the publication of this manuscript and accompanying images.

## Data Availability

The data that support the findings of this study are available from the corresponding author upon reasonable request.
